# Successful Surgical Resection for Peritoneal Implantation of Hepatocellular Carcinoma at the Paracardial Portion

**DOI:** 10.1155/2009/231854

**Published:** 2009-12-16

**Authors:** Yuichi Sanada, Shinji Osada, Yasuharu Tokuyama, Yoshihiro Tanaka, Takao Takahashi, Kazuya Yamaguchi, Kazuhiro Yoshida

**Affiliations:** Department of Surgical Oncology, Gifu Graduate School of Medicine, 1-1 Yanagido, Gifu 501-1194, Japan

## Abstract

Peritoneal implantation from hepatocellular carcinoma has been rarely reported. It may occur at various sites. Here we present a surgically resected case of peritoneal implantation to the diaphragm from hepatocellular carcinoma. A 50-year-old woman underwent right hemihepatectomy extended to a medial part of Couinaud segment IV for hepatocellular carcinoma in May 2000. In December 2008, the elevation of alpha-phetoprotein and the appearance of a heterogeneously enhanced mass, with dimensions of 9 × 7 cm, and adjacent to the remnant liver and pericardium suggested intrahepatic recurrence with markedly enhanced growth. After transcatheter arterial embolization, surgical resection under laparotomy combined with median sternotomy was selected. Samples of pericardial fluid showed no malignancy after cytological examination. At the superior border of the tumor, the confluence of pericardium and diaphragm was displaced, but the tumor itself showed a generally expanding but not invasive growth. The resected tumor showed moderately differentiated hepatocellular carcinoma whose pathology revealed a peritoneal implantation to the diaphragm. The patient is in good health without any postoperative complications or any further sign of recurrence.

## 1. Introduction

Hepatocellular carcinoma (HCC) is the most preventable type of liver carcinoma in the world. Hepatitis virus-related cirrhosis is known as the leading cause of HCC carcinogenesis. The prognosis is poor while surgery remains the best curative therapy, because the recurrence rate is quite high even after complete resection. The recurrence in almost all cases is usually detected in the remnant liver, and then the tendency is for secondaries to be found in lung or bone [1]. Therefore, peritoneal recurrence from HCC in clinical settings is rare, accounting for 2%–16% of cases [1]. Furthermore, recent reports have demonstrated that peritoneal recurrence is mainly observed on the omentum or serosal surface of the intestine [2, 3]. Because the recurrent tumor in this case appeared adjacent to the remnant liver, this solitary peritoneal recurrence of the tumor might be recognized as intrahepatic recurrence. 

Here we report a case of solitary peritoneal implantation in the diaphragm due to HCC, and the preoperative imaging, the histopathologic findings of the resected specimen, and the surgical procedure selected will be discussed with a review of the literature.

## 2. Case Report

**Figure 1:**
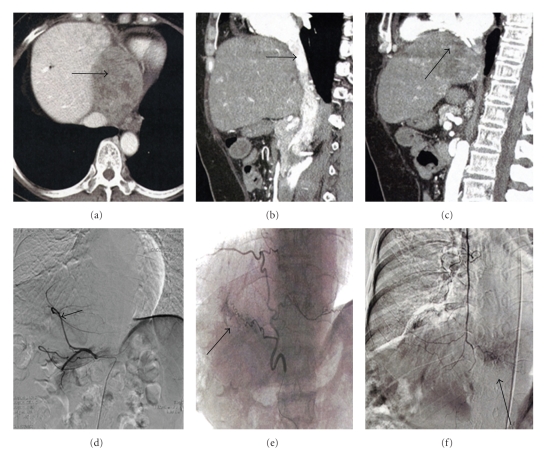
Preoperative findings. CT shows a heterogenous mass ((a)–(c), arrow), 9 cm in diameter adjacent to the remnant liver (a), displacing the IVC (b) and the pericardium (c). Angiography shows that the remnant liver is supplied by the left hepatic artery (d). Selective angiograms show a hypervascular tumor supplied from the descending branch of the inferior phrenic artery (IPA) (e) and the phrenic branch of the internal mammary artery (IMA) (f).

**Figure 2:**
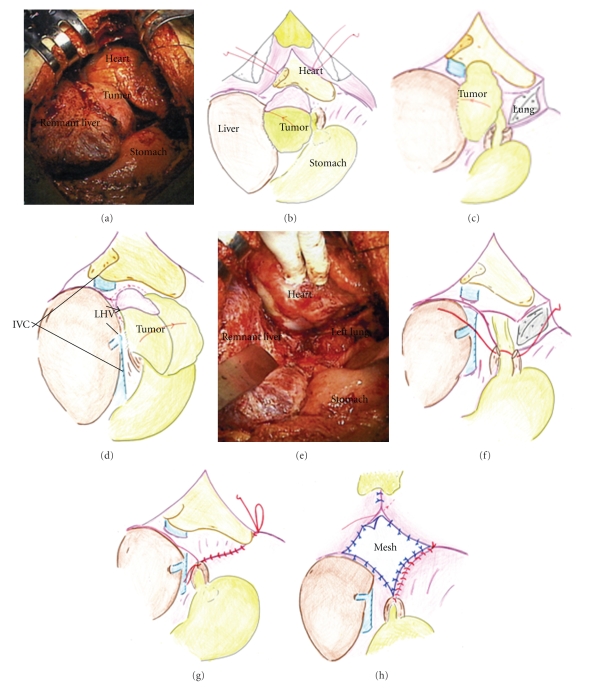
Images and schematic presentations of operative findings and procedures. By opening the pericardium, the tumor was found to be located between the remnant liver and the heart, and the diaphragm was displaced to the upper side ((a), (b)). Due to a median sternotomy with opening of the pericardium, the tumor was pulled to the right (c). The pericardium was divided from the dorsal aspect of the tumor to the left wall of the IVC (d). A complete resection can be achieved (e). Defects of the diaphragm and the pericardium were repaired ((f)–(h)).

**Figure 3:**
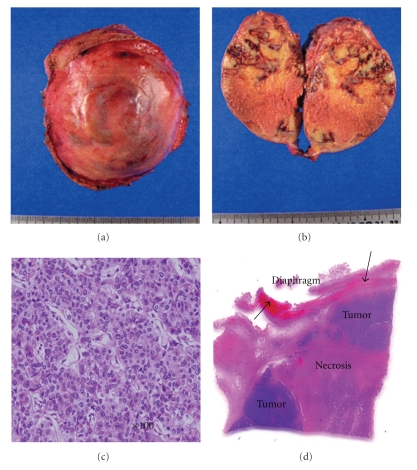
Resected specimen. The tumor is covered with the diaphragm at the cranial edge with no infiltration (a). The cut surface shows focal necrosis induced by preoperative TAE (b). The tumor is composed of moderately differentiated HCC with a thick trabecular pattern (c). The diaphragm is displaced with no infiltration to the muscular layer ((d), arrow).

A 50-year-old woman positive for hepatitis B virus underwent a right hemihepatectomy extended to a medial part of Couinaud segment IV for HCC in May 2000. The initial tumor was mainly located in Couinaud segment V and was 10 cm in diameter with capsule invasion. Histologically, it was moderately differentiated HCC with thick trabecular pattern with microscopic vascular invasion. In January 2001, partial resection of the medial segment was performed due to recurrence in the form of intrahepatic metastasis. During a period of 8 years after second surgery, no recurrence sign was detected by 5-year regular imaging follow-up at our hospital. In December 2008, the patient consulted a home doctor for chest discomfort and then was recommended to our hospital again for the purpose of general examination. The serum value of alpha-phetoprotein (AFP) increased to 248369 ng/mL (normal rage; <20 ng/mL). Computed tomography (CT) revealed a heterogenous mass, 9 × 7 cm in diameter, in the remnant liver (Figure 1(a)). The mass had expanded to the adjacent organs, inferior vena cava (IVC) (Figure 1(b)) and the pericardium (Figure 1(c)), indicating a diagnosis of intrahepatic recurrence from HCC. Angiography revealed that the remnant liver was supplied by left hepatic artery with no tumor stain (Figure 1(d)). Because selective angiograms showed a hypervascular tumor supplied from the descending branch of the inferior phrenic artery (IPA) (Figure 1(e)) and the phrenic branch of the internal mammary artery (IMA) (Figure 1(f)), transcatheter arterial embolization (TAE) was selected first. However, TAE could not relieve her chief complaint, and after evaluation that there were no other distant metastases, complete surgical resection was planned on January 2009. Because it was difficult to identify the whole aspect of the tumor from just the abdominal upper midline incision, an additional median sternotomy was carried out (Figures 2(a) and 2(b)). Pericardial fluid was found to have no malignancy after cytological examination. At the superior border of the tumor, the confluence of the pericardium and diaphragm was displaced, but the tumor itself showed general expanding growth that was not invasive. Since the adhesion on the central tendon was too complete to separate, the crus was ligated (Figure 2(c)). The diaphragm was divided toward the left edge of the sternocostal trigone, and when rotated, the heart was observed. In the opened pericardial cavity, the pericardium was transected to expose the posterior side of the tumor from the IVC (Figure 2(d)), and following transection of the posterior parietal peritoneum, the specimen was removed (Figure 2(e)). The diaphragm was sutured and the defect in the pericardium was repaired using a composix mesh (Figures 2(f)–2(h)). Macroscopically, the resected tumor, at 9.5 × 7.4 cm (Figure 3(a)), was covered with fibrous tissue (diaphragm) without evidence of infiltration and expansive necrosis was detected (Figure 3(b)). The tumor comprised moderately differentiated HCC showing a thick trabecular pattern and focal pseudogland formation (Figure 3(c)) and also the displaced muscular layer of the diaphragm was observed (Figure 3(d), arrow). These histological findings revealed a solitary peritoneal implantation to the diaphragm. The surgical resection could relieve her chest discomfort immediately. The patient is in good health without any postoperative events and no sign of recurrence.

## 3. Discussion

The recurrence from HCC is usually detected in the remnant liver and as secondary metastases in the lung or bone. In spite of the preoperative diagnosis as an intrahepatic mass, two doubts, the markedly extrahepatic growth formation and feeding routs depending on IPA and IMA, were indicated. The IPA is known as the most common source of extrahepatic collateral blood supply for HCC [4], and the IMA is demonstrated to develop as a collateral vessel after ligation of the hepatic artery [5]. And IMA enters the liver from the hepatic falciform ligament to supply the left lobe and HCCs located in S4 have been reported to feed from the right IMA [6]. In the present case, after two episodes of surgery, the arterial supply was suspected to have changed. However, both the operative findings and pathological evaluation clearly indicated that the tumor arose from the diaphragm as a solitary peritoneal recurrence. This quite rare recurrence pattern will be discussed. It is difficult to clarify that this huge tumor was a peritoneal implantation or hematogenous metastasis to the diaphragm. In the present case, the recurrent site was adjacent to the resection margin of initial hepatectomy. In addition, histologically, resected tumor was localized in the peritoneal layer of the diaphragm with no infiltration to the muscular layer of the diaphragm. Therefore, it seems reasonable to suppose that the recurrent pattern of the present case is peritoneal implantation. However, the image findings and pathologic findings in the present case remind us of the possibility for ectopic HCC in the diaphragm. Ectopic HCC is very rarely reported [7, 8]. Although we are incompetent to discuss this possibility, it is a valid assumption that this huge tumor is a peritoneal implantation because the normal liver parenchyma could not be identified throughout the resected specimen. 

The mechanism of peritoneal implantation from HCC is suggested to occur following rupture or invasion to the peritoneal cavity through the liver surface [9]. The hepatoma cells usually need a full blood supply to achieve growth, and so peritoneal implantation has been believed to be quite rare [1]. A recently published review has demonstrated that only one case of peritoneal recurrence to the diaphragm from HCC was identified out of 24 cases [2]. From the evidence that hepatitis B virus-based hepatoma related to peritoneal recurrence compared with other type of virus [10]. Li et al. pointed out that patients with HCV infection tended to be older and were characterized by more severe cirrhosis and higher incidence of tumor multicentricity [11], while Huo et al. reported that patients in the HBV group more often had a higher total tumor volume [12]. Although the incidence of peritoneal recurrence in HCC associated with HBV virus is unclear, these descriptions suggest that patients with HCC associated with the hepatitis B virus have a potential for locally exophytic growth. Accordingly, in patients with hepatitis B virus, we should pay attention to recurrent forms other than intrahepatic recurrence or distant metastasis. According to a study of 16 cases with peritoneal recurrence [10], the serosal surface of the bowel or the omentum was targeted, and there were no cases with recurrence on the diaphragm. In our patient, two episodes of surgery might have supported a full blood supply to hepatoma cells on the diaphragm during the stages of healing.

It has been reported that the 1-, 3-, and 5-year survival rates after resection of peritoneal recurrence of HCC were 62.5%, 34.1%, and 30.1%, respectively, and the presence of capsule invasion was found to be the most significant prognostic factor [10]. However, due to the rarity of this recurrent form there have been no studies of the postoperative prognosis between peritoneal recurrence and other recurrent forms. Poon et al. [13] reported that an aggressive strategy is likely to be more favorable for patients with solitary extrahepatic recurrence. In the present case, fortunately, the peritoneal tumor with capsule was detected as solitary and developed expansively to the diaphragm. According to the usual interval for the appearance of peritoneal recurrence, which ranges from 0 to 84 months [2], the present case is a notable long-term recurrence after the initial surgery. The long interval between initial surgery and recurrence in the present case suggests the low malignant behavior. Nakayama et al. reported that resection of peritoneal recurrence arising from HCC may be of value in improving patient survival [14]. However, it is still unclear whether this variation in the interval of peritoneal recurrence has different implications for prognosis after resection. Due to disappointing outcomes from chemotherapy for HCC [15], it is necessary to diagnose new tumors by regular examination and to treat them with aggressive surgical procedures.
